# Docking guided phase display to develop fusion protein with novel scFv and alkaline phosphatase for one-step ELISA salbutamol detection

**DOI:** 10.3389/fmicb.2023.1190793

**Published:** 2023-05-12

**Authors:** Shuai Hu, Guangbo Yang, Zhou Chen, Qiuye Li, Bin Liu, Ming Liu, Dawei Zhang, Shan Chang, Ren Kong

**Affiliations:** ^1^Institute of Bioinformatics and Medical Engineering, School of Electrical and Information Engineering, School of Chemical and Environmental Engineering, Jiangsu University of Technology, Changzhou, China; ^2^Beijing New BioConcepts Biotech Co., Ltd., Beijing, China

**Keywords:** phage display, docking, salbutamol, scFv, ELISA

## Abstract

**Introduction:**

Salbutamol (SAL) is a β2 adrenergic receptor agonist which has potential hazardous effects for human health. It is very important to establish a sensitive and convenient method to monitor SAL.

**Methods:**

Here we introduce a method to combine the information from docking and site specific phage display, with the aim to obtain scFv with high affinity to SAL. First, single chain variable fragment (scFv) antibodies against SAL were generated through phage display. By using molecular docking approach, the complex structure of SAL with antibody was predicted and indicated that H3 and L3 contribute to the binding. Then new libraries were created by randomization specific residues located on H3 and L3 according to the docking results.

**Results and discussion:**

Anti-SAL scFv antibodies with high efficiency were finally identified. In addition, the selected scFv was fused with alkaline phosphatase and expressed in *E coli* to develop a rapid and low-cost one step ELISA to detect SAL.

## Graphical Abstract

The flowchart for discovery of anti-SAL scFvs. By using molecular docking approach and phage display, anti-SAL scFvs with high affinity were identified and fused with alkaline phosphatase for one-step ELISA salbutamol detection. 
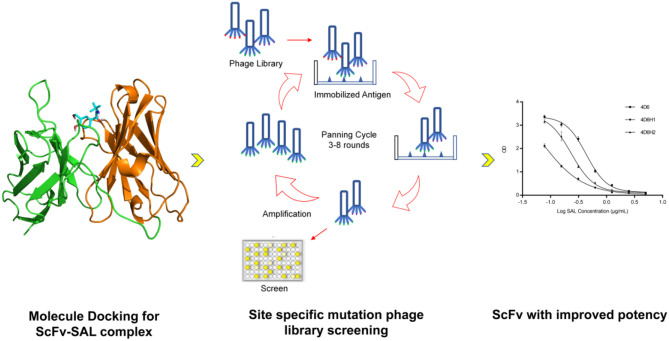

## 1. Introduction

Salbutamol (SAL) is a β2 adrenergic receptor agonist, which is widely used to treat bronchial asthma (Price and Clissold, 1989). Meanwhile, it can promote protein synthesis, increase animal lean meat rate, and improve feed conversion rate. It is often illegally used as a feed additive in animal husbandry (Baker et al., 1984; Dalrymple et al., 1984; Jones et al., 1985). Excessive intake of SAL can cause myalgia, headache, dizziness, nervousness, tachycardia, nausea, vomiting, and even cause liver and kidney damage, and its residues pose a serious hazard to human health (Wang and Shen, 2007; Khamta et al., 2009; Sheu et al., 2009). Therefore, SAL has been strictly banned as a feed additive by many countries, but due to its economic incentives, many farms still use SAL extensively (Kearns et al., 1985; Garssen et al., 1995). Illegal addition of SAL can cause environmental pollution and affect public health via the food chain (Wang et al., 2015). Studies have shown that SAL has the possibility of entering the ecological environment through animal feces and urine. While causing environmental pollution, it then enters the human body through indirect channels (Fang et al., 2019). SAL has already been a widespread environmental pollutant (Depaolini et al., 2016). At present, SAL residues have been found in natural waters around the world, including tap water, wastewater, treated sewage, and river water (Yamini et al., 2006; Lei et al., 2015a). Although the concentration of SAL in some water bodies has reached 470 ng/L (Bound and Voulvoulis, 2006), there are few reports focusing on environmental problems caused by SAL (Liu et al., 2018). Therefore, it is imperative to establish a sensitive method to monitor SAL.

The analytical methods currently used to detect SAL include gas chromatography–mass spectrometry (GC-MS) (Black and Hansson, 1999), high-performance liquid chromatography (HPLC) (Rosales-Conrado et al., 2013), and high-performance liquid chromatography–mass spectrometry (HPLC-MS) (Zhang et al., 2012). Because these methods require cumbersome sample preparation before instrumental analysis (Liu Z. J. et al., 2016), it is difficult to meet the requirements for high-throughput and rapid screening of a large number of environmental samples. Immunoassay is a fast, low-cost, and high-throughput method, and it is becoming a reliable tool for the analysis of environmental pollutant residues. So far, many immunoassays for detecting SAL have been successfully developed. Among them, ELISA is the commonly used method for SAL detection (Degand et al., 1993; Lei et al., 2008, 2015b). Chemiluminescence and electrochemiluminescence assay, time-resolved immunofluorescence technique, and lateral chromatography technique (colloidal gold) have been developed for SAL and other β-agonist detection (Cai et al., 2015; Xu et al., 2015, 2022; Liu B. et al., 2016; Li et al., 2017; Gu et al., 2020).

Immunoassay methods also have some problems. For example, most of the currently available anti-SAL antibodies are polyclonal antibodies from sheep and rabbits (Degand et al., 1993; Lei et al., 2008; Wu et al., 2014), and their specificity is usually poor. For polyclonal antibodies, the heterogeneity of antibody preparations usually leads to cross-reactions with highly similar antigens. For example, polyclonal anti-SAL antibody shows significant cross-reactivity to clenbuterol (Degand et al., 1993; Lei et al., 2008; Wu et al., 2014). Compared with polyclonal antibodies, monoclonal antibodies have the advantages of high specificity, and a relatively simple production process has been widely used in immunoassays. Adam et al. obtained an anti-salbutamol antibody by immunizing mice in 1990 and used the antibody to develop a radioimmunoassay for detecting salbutamol (Adam et al., 1990). Xie et al. (2012) developed a new monoclonal anti-SAL antibody with improved affinity. Monoclonal antibodies have some disadvantages when applied in an immunoassay, such as high cost and long production cycle. ScFv antibodies can be expressed using *Escherichia coli*, effectively addressing the abovementioned issues. A chicken scFv anti-SAL has also been isolated and characterized by Lee et al. (2018). However, the initial affinity of these antibodies is typically too low for further application. High affinity and selectivity are critical issues for detecting ability so it is necessary to improve the affinity of antibodies derived from phage libraries.

Since antibodies still play a key role in immunoassays, the use of high-affinity antibodies could significantly improve the level of detection. Here, we have obtained anti-SAL scFvs from a rationally designed synthetic antibody library and improved its binding capacity with SAL through affinity maturation. In particular, the scFv binding site for SAL was predicted by molecular docking, and then, point mutation libraries were constructed based on the predicted results, which were used for antibody affinity maturation (Graphical Abstract). In addition, the scFv was fused with alkaline phosphatase (scFv-AP) which could directly react with pNPP for colorimetric development without the use of secondary antibodies so that the detection process was simplified. At the same time, the scFv-AP can be expressed in *E. coli* on a large scale, which further reduces the cost.

## 2. Materials and methods

### 2.1. Synthetic antibody library

The library used in this study was provided by BioNC (www.bionc.com.cn), and it contained over 3 × 10^10^ human antibodies in the scFv format. The library was designed with the aim to obtain highly stable antibody clones, which were found to be highly functional, as ~80% of randomly selected clones expressed the corresponding antibody.

### 2.2. Biopanning

Biopanning of anti-SAL scFv from constructed phage-displayed scFv library was carried out according to standard protocols with some modifications (Barbas et al., 2001). In brief, in the first round of biopanning, immunotubes were coated with 100 μg/ml of SAL-BSA in carbonate coating buffer overnight at 4°C and washed six times with PBST (PBS containing 0.1% Tween20). After being blocked with 5% skim milk for 1 h at 37°C and washed 10 times, ~10^11^ pfu/ml recombinant phages were added into SAL-BSA coated immunotubes and incubated for 1 h at 37°C with gentle shaking. The unbound phages were washed away with PBST 20 times. Then, the bound phages were eluted with elution buffer (0.2 M Glycine-HCl pH2.2) for 10 min at 37°C and immediately neutralized with 1 M Tris–HCl (pH9). In total, 500 μl of log phase *E. coli* TG1 was added to tubes and reinfected at 37°C for 1 h. The infected cells were plated on an LB agar plate containing 100 μg/ml ampicillin and 2% glucose and incubated overnight at 37°C. For the next three rounds of biopanning, in all cases, the number of inputted phages remained the same while the concentration of coated SAL-BSA/OVA/KLH was gradually reduced to 75, 50, and 25 μg/ml in the second, third, and fourth rounds of biopanning, respectively.

### 2.3. Selection of recombinant phage antibody clones

Phage ELISA was carried out to identify phages that can specifically bind with SAL. In brief, microtitration plates (Corning) were coated with 1 μg/ml SAL-BSA or BSA (negative control) and blocked with 5% skim milk. Phage solution diluted with equivalent blocking buffer was added to the plate and incubated for 1 h at 37°C. After washing five times with PBST, 100 μl of HRP-conjugated anti-M13 antibody diluted 1:5,000 in PBST to detect bonded recombinant phage scFv was added and incubated for 1 h at 37°C. After washing as mentioned above, 100 μl of TMB was added to each well for colorimetric development, and the reaction was stopped with 2 M H_2_SO_4_. The absorbance was determined at 450 nm with a microtiter plate reader (TECAN). Afterward, ELISA-positive phage clones were used for sequencing.

### 2.4. Competitive monoclonal phage ELISA

Competitive phage ELISA was performed to determine the specificity of the isolated phage-scFvs toward SAL. Selected clones with the highest apparent affinity were further characterized by multipoint competitive phage ELISA. Phages were normalized to 5 × 10^9^ cfu and challenged by incubation with SAL standards ranging from 0 to 10.0 μg/ml in 5 μg/ml SAL-BSA-coated microtitration plates in the first incubation step. Plates were washed with PBST three times and added with 1:5,000 HRP-conjugated anti-M13 antibody. Then, plates were washed with PBST three times, the colorimetric development was performed by the addition of TMB, the reaction stopped with 2M H_2_SO_4_, and the absorbance was measured at 450 nm.

### 2.5. Construction of phage library with CDR randomization

Two libraries were constructed for sequential randomization of CDR-H3 and CDR-L3, using degenerated oligonucleotides with the NNS motif (N = A/C/G/T and S = C/G) (Barbas et al., 2001). The first library was constructed by mutating the five residues at CDR-H3 located at positions 99–103. The second library was prepared to randomize CDR-L3 residues 213–221 of the CDR-H3 mutant. The oligonucleotides were designed to permit any amino acid at each position while decreasing the presence of stop codons and cysteines. PCRs were performed by using the KOD polymerase (Takara).

### 2.6. Cloning, expression, and purification of anti-SAL scFv/scFv-AP

The selected scFv genes were amplified and ligated into pET-22b (+) and AP expression vector bAP (BioNC) by seamless cloning using a pEASY-Basic Seamless Cloning and Assembly kit (TransGen), according to the manufacturer's instructions. The ligated plasmids were heat-shocked and transformed into *E. coli* TG1 competent cells. Transformed cells were plated onto LB agar plates with 100 μg/ml ampicillin and incubated overnight at 37°C. Colony PCR was used to screen for positive clones harboring scFv inserts. Afterward, correct clones were used for sequencing. The sequenced plasmids were transformed into *E. coli* BL21(DE3) competent cells, which were grown in 100 ml LB medium containing 100 μg/ml ampicillin with shaking at 37°C until the OD600 reached 0.8. Isopropyl-β-D-thiogalactopyranoside (IPTG) with a final concentration of 0.6 mM was added, and the bacteria were allowed to grow overnight at 25°C with continuous shaking at 220 rpm. The cell pellets were harvested and thoroughly re-suspended in sonication buffer (20 mM Tris, 300 mM NaCl, pH 8.0), containing protease inhibitor (Sigma) and DNase I (Sango), according to the manufacturer's instructions. Then, the re-suspended cell pellets were lysed by sonication on the ice at 300W for 30 min (sonication for 4 s and intermission for 6 s) using a 6 mm diameter microtip (Scientz). The resulting suspension was centrifuged at 12,000 *g* for 30 min at 4°C. The supernatant was then applied to a 5 ml Ni-Sepharose column equilibrated with buffer A (20 mM Tris, 300 mM NaCl, and 10 mM imidazole, pH 8.0). The column was washed first with buffer A and then with buffer B (20 mM Tris, 300 mM NaCl, and 20 mM imidazole, pH 8.0). Protein was eluted with Buffer C (20 mM Tris, 300 mM NaCl, and 200 mM imidazole, pH 8.0). Purified proteins were analyzed by SDS–PAGE.

### 2.7. ELISA for measuring the binding of scFv and scFv-AP with SAL

ELISA was performed to measure the binding of the expressed scFv and scFv-AP with SAL. Each well of the microtitration plates (Corning) was coated with 100 μl of 1 μg/ml SAL-BSA in PBS and incubated for 1 h at 37°C followed by blocking with 200 μl of 5% skim milk for 1 h at 37°C. A total of 100 μl purified scFv or scFv-AP, which has been mixed with various concentrations of free SAL for 1 h was added to the plate and incubated for another 1 h at 37°C. For the scFv added wells, after washing with PBST, 100 μl of 1:5,000 diluted HRP-conjugated Anti-His Tag antibody (BioNC) was added to 5% skim milk, and the plate was incubated for 1 h at 37°C. After a second wash with PBST, 100 μl of TMB was added, and the OD was measured at 450 nm. Meanwhile, for the scFv-AP added wells, after washing with PBST, 100 μl of pNPP was added, and the OD was measured at 405 nm.

### 2.8. Homology modeling and SAL docking

The 3D structure of 4D6 scFv was predicted by Robetta with default parameters (https://robetta.bakerlab.org/). AutoDock Vina is used as a docking tool to explore the binding of SAL with 4D6 (Trott and Olson, 2010). The built model of 4D6 is used as a receptor and prepared by MGLTools to add hydrogens and Kollman charge. The docking box center is set to the geometric center of CDR loops, and the box size is set to 28 × 28 × 8 Å, containing the entire CDR domains. The three-dimensional structure of SAL in the sdf format is downloaded from PubChem (https://pubchem.ncbi.nlm.nih.gov/compound/Salbutamol) and OpenBabel (O'Boyle et al., 2011) and is used to convert it into a mol2 file. The MGLTools are used to generate the ligand pdbqt file with Gasteiger charge added and flexible bond set as default. The Vina was used to dock SAL to 4D6 with all the other parameters set as defaults.

## 3. Results

### 3.1. Phage displaying of ScFv antibody, biopanning, and screening

**Figure 1:**
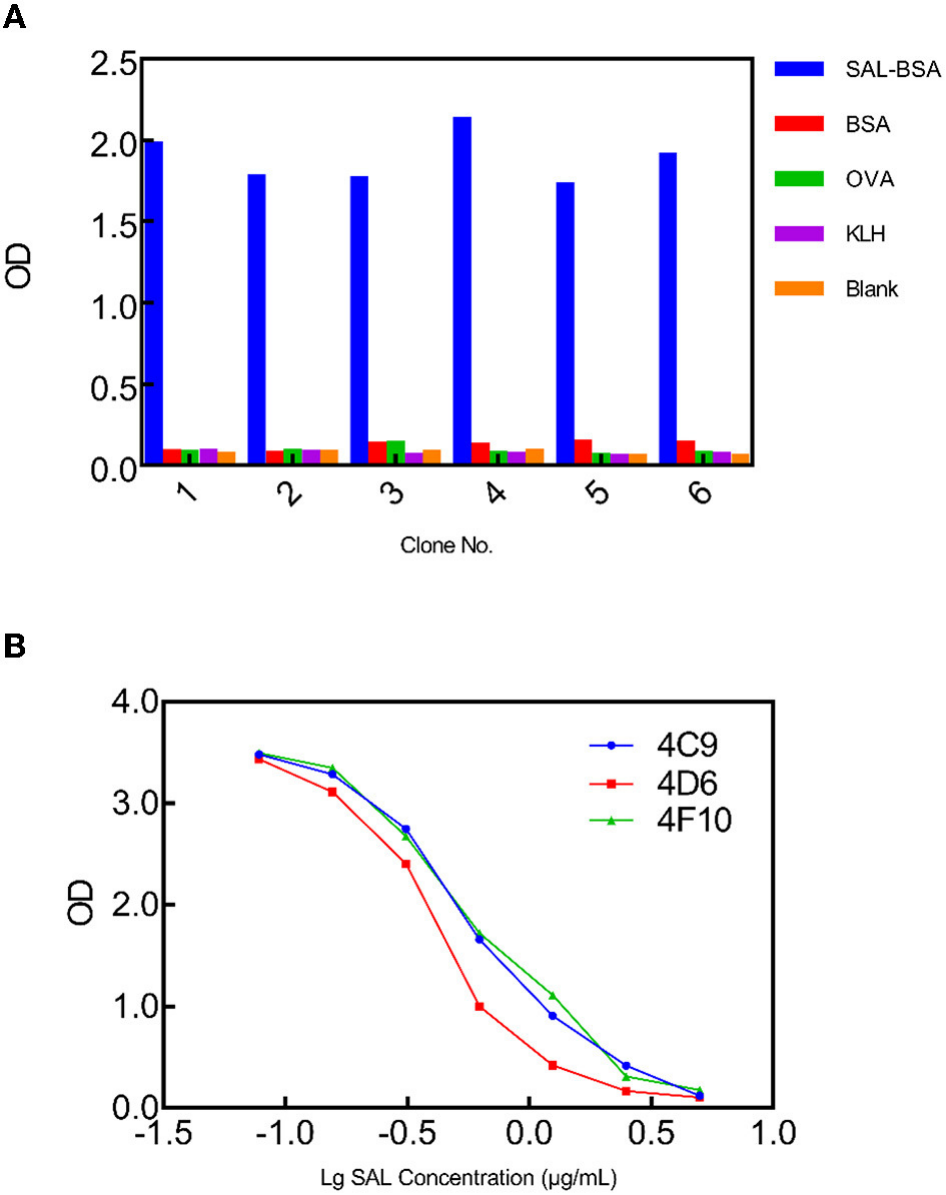
Phage ELISA to select SAL-specific clones. **(A)** Monoclonal phage ELISA titers from the synthetic library. SAL-specific phage clones were isolated from output 4. Values indicated are means from duplicate readings. **(B)** Multipoint competitive phage ELISA for the selected clones 4C9, 4D6, and 4F10 indicating concentration-dependent inhibition. Values indicated are mean absorbances from duplicate readings.

The library glycerol stock was inoculated into the LB medium and grown at 37°C. In the presence of helper phage M13KO7, the scFv fusion protein was displayed on the surface of the recombinant phage, allowing for biopanning and affinity screening. A biopanning strategy was devised in which the SAL carrier protein conjugates would be different for each panning round. The aim of the panning strategy was to reduce scFvs which bound the carrier protein rather than SAL itself. SAL-BSA was used in the first and last rounds of panning, while SAL-OVA and SAL-KLH were used in the second and third rounds, respectively. SAL-BSA-specific phages were obtained after the fourth panning round and showed no binding toward any of the irrelevant carrier proteins based on the polyclonal phage ELISA results (Figure 1A). To further discern which of these clones were specific to SAL alone, competitive monoclonal phage ELISA was carried out. From this experiment, three clones from the fourth panning round were found to be specific to SAL (Figure 1B). Sequencing results showed that they contained different scFv fragments. Since clone 4D6 exhibited the highest affinity with SAL, it was chosen for the next improvement by affinity maturation.

### 3.2. Interactive docking of SAL to 4D6

**Figure 2:**
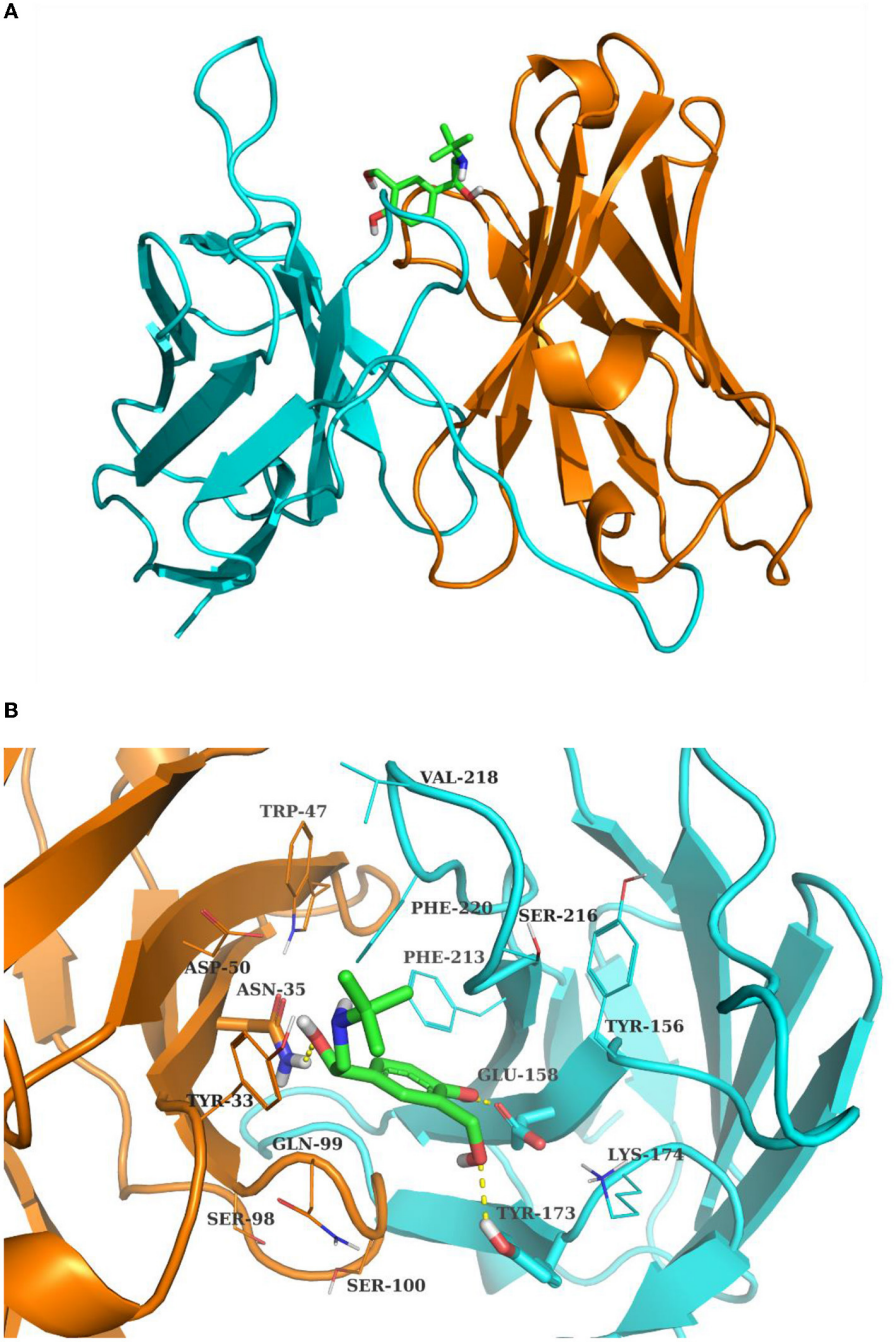
The complex structure of SAL and 4D6 predicted by Autodock Vina. **(A)** Total view; **(B)** detailed view. The H chain and L chain are shown in the cyan and orange cartoon model, respectively. SAL is shown in the sticks model. The contact residues are depicted in lines, and residues hydrogen bonding with SAL is shown in the sticks model. The hydrogen bonds are drawn by yellow dashed lines.

The 4D6-SAL complex model was predicted by AutoDock vina with the standard docking protocol. Amino acids are numbered sequentially. A total of 10 poses are generated with docking scores ranging from −5.961 to −5.362 kcal/mol. For the top one docking pose, SAL bound between the H chain and L chain, forming contacts with residues from CDR loops (Figure 2A). Hydrogen bonds were observed between the hydroxyl groups of SAL and side chains of ASN35, GLU158, and TYR173 (Figure 2B). Otherwise, SAL formed interactions with TYR33 from CDR-H1; TRP47 from FR-H2; ASP50 from CDR-H2 and GLN99; SER100 from CDR-H3; TYR156 and LYS174 from FR-L2; PHE213, SER216, VAL218, and PHE220 from CDR-L3. In this model, HCDR3 and LCDR3 of the 4D6 scFv appeared to contribute mainly to antigen binding.

### 3.3. CDR-H3 and CDR-L3 affinity maturation by random mutagenesis

**Figure 3:**
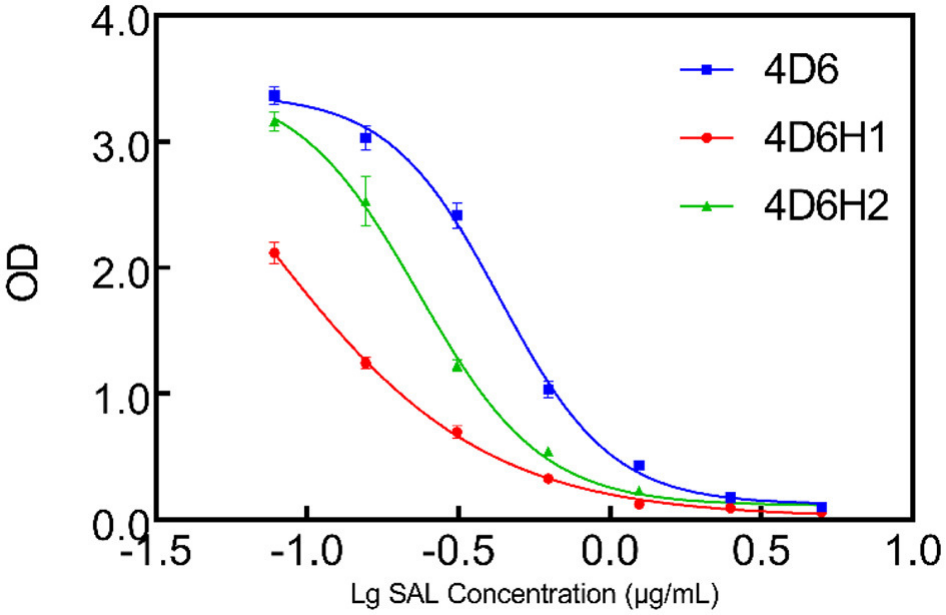
Competition ELISA for measuring relative affinities of anti-SAL scFvs by CDR-H3 maturation. The inhibition of anti-SAL scFv binding to SAL was analyzed on SAL-BSA-coated plates with different concentrations of free SAL, and the curves were fitted to a four-parameter model using Prism7 software.

The CDR-H3 and CDR-L3 affinity maturation of 4D6 scFv was performed by *in vitro* evolution. The use of degenerated oligonucleotides reduces the number of oligonucleotides and introduces a natural evolution in the process (Yang et al., 1995). According to the docking results, the four amino acids between positions 99–103 in CDR-H3 were selected to randomize by using a phage scFv library of 1 × 10^6^ transformants. Two scFvs, 4D6H1 and 4D6H2, with improved recognition of SAL by ELISA, were obtained after three rounds of panning. Analysis by ELISA showed that the efficiency of 4D6H1 and 4D6H2 was 0.066 and 0.23 μg/ml, having ~seven-fold and ~two-fold improvement with respect to the parental 4D6 scFv (0.43 μg/ml), respectively (Figure 3 and Table 1). The most improved variant (4D6H1) presented two amino acid mutations (T101S and F103Y; Table 1). For the 4D6H2 scFv, we observed three different mutations (T101A, G102D, and F103T).

**Table 1:** Mutations of anti-SAL clones derived from the random CDR-H3 and CDR-L3 libraries.

**Clone**	**CDR-H3**					**CDR-L3**									**IC_50_ (μg/ml)**	Efficiency increase^*^
	**99**	**100**	**101**	**102**	**103**	**213**	**214**	**215**	**216**	**217**	**218**	**219**	**220**	**221**		
4D6	Q	S	T	G	F	F	Q	G	S	N	V	P	F	T	0.43	1
4D6HI	Q	S	**S**	G	**Y**	F	Q	G	S	N	V	P	F	T	0.066	~7
4D6H2	Q	S	**A**	**D**	**T**	F	Q	G	S	N	V	P	F	T	0.23	~2
4D6H1L1	Q	S	S	G	Y	F	Q	G	**D**	N	**T**	P	F	T	0.055	~9
4D6H1L2	Q	S	S	G	Y	F	Q	G	**N**	N	**T**	P	F	**L**	0.017	~25

* The efficiency increase is a relative value to 4D6. The bold letters indicate the mutated residues.

**Figure 4:**
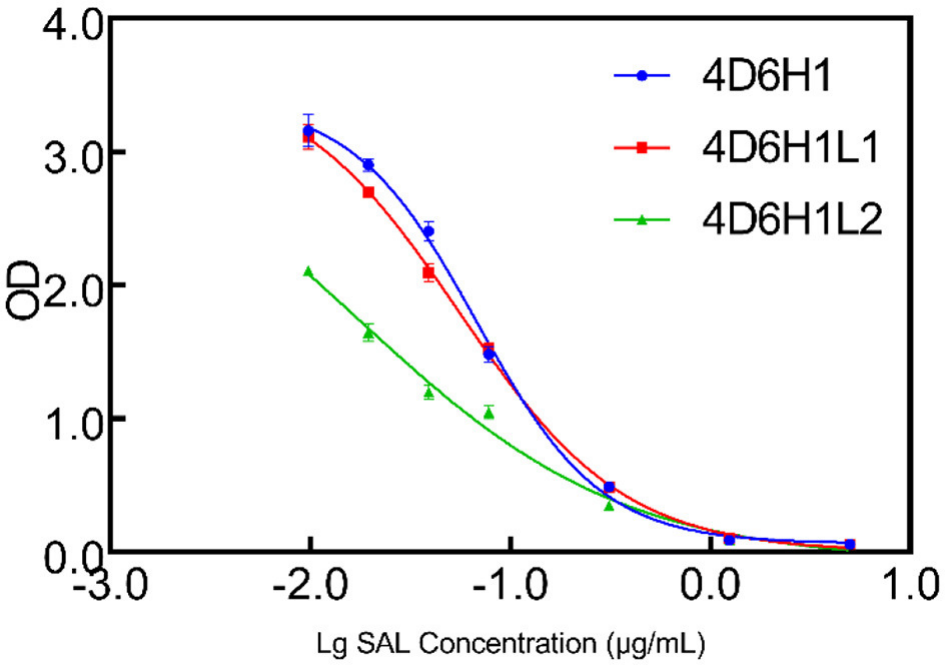
Competition ELISA for measuring relative affinities of anti-SAL scFvs by CDR-L3 maturation. The inhibition of anti-SAL scFv binding to SAL was analyzed on SAL-BSA-coated plates with different concentrations of free SAL, and the curves were fitted to a four-parameter model using Prism7 software.

4D6H1 scFv was selected to initiate the second round of maturation based on CDR-L3 mutagenesis. Based on the docking results, a new library was created by randomization of the four amino acids located at positions 213–221. The resulting phage library contained 1.5 × 10^6^ different transformants. Two scFvs, 4D6H1L1 and 4D6H1L2, were obtained after three rounds of panning. It is shown that 4D6H1L2 had mostly efficient recognition of SAL with IC_50_ of 0.017 μg/ml, approximately a four-fold improvement to 4D6H1 (Figure 4). The amino acid mutations are presented in Table 1.

### 3.4. Soluble scFv/scFv-AP expression and analysis

**Figure 5:**
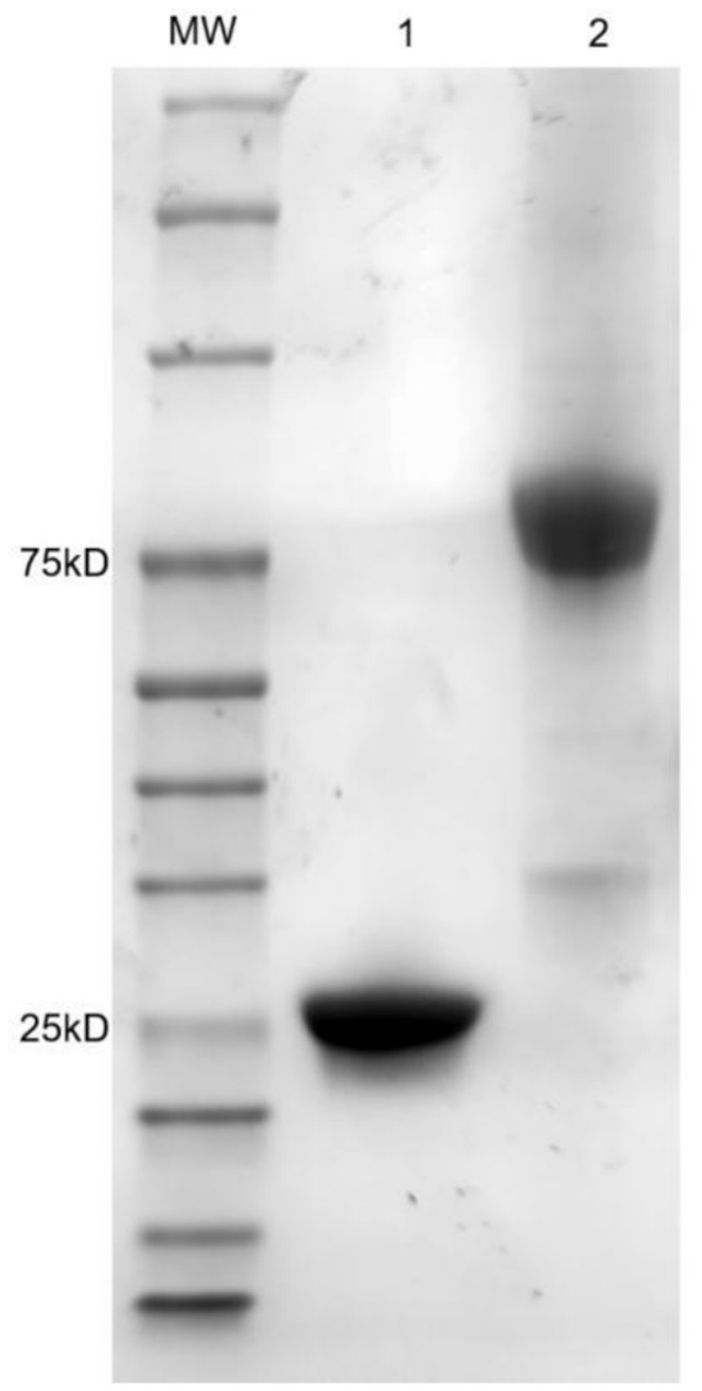
SDS–PAGE analysis of purified scFv and scFv-AP fusion protein. Lane 1: 4D6HIL2 scFv; Lane 2: 4D6HIL2-AP; MW: molecular weight markers in kDa are indicated on the left.

**Figure 6:**
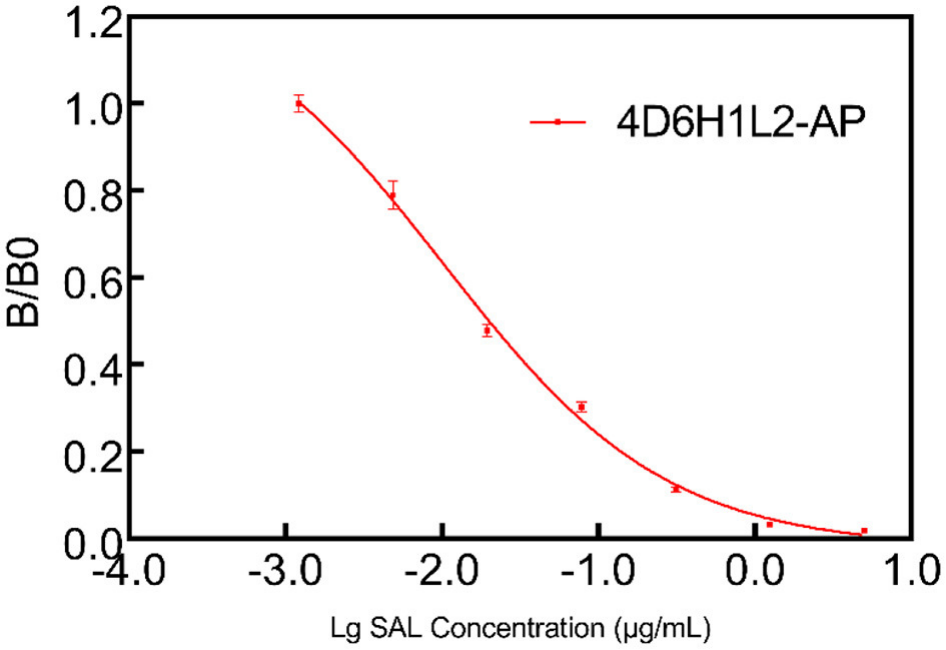
Multipoint competitive ELISA to evaluate the functionality of the scFv-AP conjugate. The 4D6HIL2-AP showed good efficiency to SAL in the one-step ELISA test.

4D6H1L2 scFv gene fragment was seamlessly cloned into the pET-22b (+) vector and the AP expression vector bAP. Then, the correctly sequenced recombinant plasmid was transformed into BL21 (DE3). After IPTG induction, the scFv and scFv-AP were purified through Ni-NTA affinity chromatography. The size and purity of scFv were verified on SDS–PAGE gel with one major band shown at MW ~25 kD. Meanwhile, the scFv-AP fusion protein showed one dominant band with an expected size of ~75 kD by SDS–PAGE (Figure 5). The binding activity of 4D6H1L2 scFv-AP to SAL was evaluated in competitive ELISA (Figure 6). The scFv-AP can serve as a bifunctional immunoreagent integrating recognition of SAL with high enzymatic activity. The IC_50_ value of the scFv-AP for SAL is 0.010 μg/ml, comparable to 4D6H1L2 scFv alone, 0.017 μg/ml. It is indicated that the fusion protein keeps the same or even a little bit better ability to recognize SAL in the assays. Otherwise, the dimerization of the fusion protein might improve the binding avidity of scFv, further giving rise to the improvement of sensitivity to SAL (Kortt et al., 2001; Liu et al., 2013).

## 4. Discussion

Antibodies are still important molecular probes for detecting β2 agonists in biological and environmental matrices. There have been many studies using phage display technology to produce specific antibodies against various antigens. In this study, specific anti-SAL scFvs were screened from a synthetic antibody library. It is important to perform panning with blocking reagents before positive selection, which will significantly reduce the number of non-specific phages. Meanwhile, switching the carrier protein between each panning round reduces the presentation frequency of non-specific motifs of the carrier protein itself.

Clone 4D6 showed the strongest binding affinity based on phage ELISA (Figure 1B), hence, we performed a stepwise *in vitro* affinity maturation procedure to improve the affinity of 4D6. We obtained a 3D model of the 4D6-SAL complex to identify the amino acids in the CDRs that needed to be mutated. In this model, CDR-H3 and CDR-L3 seemed to be involved in SAL binding. The heavy chain is considered to contribute to antigen binding to a greater extent than the light chain, especially through the CDR-H3 (Chothia and Lesk, 1987). Therefore, mutagenesis in the CDR-H3 was attempted first. We identified two clones, 4D6H1, which had a seven-fold increased affinity, and 4D6H2, which had a two-fold increased affinity. The differences in the affinities of 4D6H1 and 4D6H2 might be caused by the changes in different amino acids. The mutations introduced in 4D6H1 might have contributed to the better positioning of the residues for tighter binding to SAL than the mutations in 4D6H2. Then, we performed randomization of the CDR-L3 using 4D6H1 as the template. Two clones, 4D6H1L1 and 4D6H1L2, were identified from the CDR-L3 randomized library, with an ~25-fold improvement compared to the parental clone 4D6. This phenomenon was consistent with numerous structural and functional studies, which have shown that HCDR3 usually plays a dominant role in antigen binding (Xu and Davis, 2000; Martinez et al., 2007; Schoonbroodt et al., 2008). Finally, we selected 4D6H1L2 for further characterization because of its highest binding affinity.

The level of sensitivity of the immunoassay depends on a number of factors, especially the affinity of detecting antibodies and the enzyme used. Varieties of enzymes are used as markers in immunoassays, and the most commonly conjugated enzymes are horseradish peroxidase, alkaline phosphatase, β-galactosidase, and glucoamylase (Wild, 2013). AP is frequently used due to its number of advantages, such as its high catalytic activity, good enzymatic stability, high affinity and high turnover for a large range of substrates, and easy conjugation to antibodies in a cost-efficient way (Chen et al., 2018).

Here, we choose AP to develop a fusion protein with the scFv 4D6H1L2, with the aim to detect SAL by one-step ELISA. Traditional ELISAs for SAL are commonly performed in a two-step competitive protocol consisting of a primary antibody and a secondary antibody conjugated to HRP. Compared with traditional ELISAs, less immunoreagents and shorter time were required in the scFv-AP-based ELISA because the addition of a secondary antibody was avoided. It is shown that scFv-AP presents high efficiency to detect SAL in one-step ELISA, with IC_50_ of 0.010 μg/ml (Figure 6). Since the IC_50_ value of salbutamol detected by 4D6H1L2 scFv was 0.017 μg/ml, this indicates that the fusion of AP did not lead to a decrease in antibody affinity. In addition, the fusion protein could be easily produced and expressed in bacteria with a high yield which could reach 300 mg/L (data not shown). Furthermore, to the best of our knowledge, this study is the first to apply AP fusion scFv antibody for the detection of SAL. Moreover, the sensitivity of scFv-AP-based ELISA is good enough to detect SAL in environmental matrices. The resulting assay showed great promise for rapid analysis of SAL in various samples, especially in the case of a large screening campaign, reducing the analysis time and the cost of analysis.

## 5. Conclusion

A novel scFv antibody (4D6) against the β2-agonist SAL from a synthetic antibody library was successfully isolated and characterized. Molecular docking is used to predict the complex structure of 4D6 and SAL. The model shows that residues on H3 and L3 contribute to the binding of SAL. Then, the CDR-H3 and CDR-L3 affinity maturation of 4D6 scFv was performed by *in vitro* evolution, and 4D6H1L2 was finally identified with high efficiency to detect SAL. The fusion protein is constructed by 4D6H1L2 and alkaline phosphatase, which is applied to one-step ELISA and showed good ability for SAL detection. The study may provide a new method for rapid analysis of SAL in various samples.

## Data availability statement

The original contributions presented in the study are included in the article/Supplementary material, further inquiries can be directed to the corresponding authors.

## Author contributions

SH, GY, SC, ML, and RK: conceptualization. SH, GY, and RK: methodology. SH, DZ, SC, and RK: software. SH, GY, ZC, BL, and RK: formal analysis and writing—original draft preparation. SH, GY, BL, ML, QL, DZ, SC, and RK: investigation. ML, ZC, and SC: resources. SH, GY, DZ, QL, and RK: data curation. SH, GY, ZC, ML, QL, and SC: writing—reviewing and editing. SC, ML, and RK: supervision. All authors have read and agreed to the published version of the manuscript.
