# Supplementary material for: Docking guided phase display to develop fusion protein with novel scFv and alkaline phosphatase for one-step ELISA salbutamol detection
Source: Front Microbiol. 2023 May 12;14:1190793. doi: 10.3389/fmicb.2023.1190793 (PMC10213401; doi:10.3389/fmicb.2023.1190793)
Supplement: Supplementary file 1 [file Data_Sheet_1.docx]

| Pose ID | docking score  (kcal/mol) |
| --- | --- |
| 1 | -5.961 |
| 2 | -5.945 |
| 3 | -5.846 |
| 4 | -5.777 |
| 5 | -5.769 |
| 6 | -5.726 |
| 7 | -5.706 |
| 8 | -5.635 |
| 9 | -5.487 |
| 10 | -5.362 |

**Supporting table 1.** The docking score of ten poses generated by AutoDock vina.

The sequence of 4D6: QVQLQQSGPELVKPGASVKISCKASGYTFTDYYMNWVKQSHGKSLEWIGDINPNNGGTSYNQKFKGKATLTVDKSSSTAYMELRSLTSEESSVYYCESQSTGFWGQGTLVTVSAGGGGSDILMTQTPSSLPVSLGDQASISCRSSQSIVHSNGNTYLEWYLQKPGQSPKLLIYKVSNRFSGVPDRFSGSGSGTDFTLKISRVEAEDLGVYYCFQGSNVPFTFGSGTKLEIK

The sequence of 4D6H1：

QVQLQQSGPELVKPGASVKISCKASGYTFTDYYMNWVKQSHGKSLEWIGDINPNNGGTSYNQKFKGKATLTVDKSSSTAYMELRSLTSEESSVYYCESQSSGYWGQGTLVTVSAGGGGSDILMTQTPSSLPVSLGDQASISCRSSQSIVHSNGNTYLEWYLQKPGQSPKLLIYKVSNRFSGVPDRFSGSGSGTDFTLKISRVEAEDLGVYYCFQGSNVPFTFGSGTKLEIK

The sequence of 4D6H2：

QVQLQQSGPELVKPGASVKISCKASGYTFTDYYMNWVKQSHGKSLEWIGDINPNNGGTSYNQKFKGKATLTVDKSSSTAYMELRSLTSEESSVYYCESQSADTWGQGTLVTVSAGGGGSDILMTQTPSSLPVSLGDQASISCRSSQSIVHSNGNTYLEWYLQKPGQSPKLLIYKVSNRFSGVPDRFSGSGSGTDFTLKISRVEAEDLGVYYCFQGSNVPFTFGSGTKLEIK

The sequence of 4D6H1L1：

QVQLQQSGPELVKPGASVKISCKASGYTFTDYYMNWVKQSHGKSLEWIGDINPNNGGTSYNQKFKGKATLTVDKSSSTAYMELRSLTSEESSVYYCESQSSGYWGQGTLVTVSAGGGGSDILMTQTPSSLPVSLGDQASISCRSSQSIVHSNGNTYLEWYLQKPGQSPKLLIYKVSNRFSGVPDRFSGSGSGTDFTLKISRVEAEDLGVYYCFQGDNTPFTFGSGTKLEIK

The sequence of 4D6H1L2:

QVQLQQSGPELVKPGASVKISCKASGYTFTDYYMNWVKQSHGKSLEWIGDINPNNGGTSYNQKFKGKATLTVDKSSSTAYMELRSLTSEESSVYYCESQSSGYWGQGTLVTVSAGGGGSDILMTQTPSSLPVSLGDQASISCRSSQSIVHSNGNTYLEWYLQKPGQSPKLLIYKVSNRFSGVPDRFSGSGSGTDFTLKISRVEAEDLGVYYCFQGNNTPFLFGSGTKLEIK
